# Tumefaction of the Parotid Region in a 10-Month-Old Infant Revealing a Synovial Sarcoma: A Diagnostic Challenge, Case Report, and Review of the Literature

**DOI:** 10.7759/cureus.102268

**Published:** 2026-01-25

**Authors:** Hind Rachidi, Fatima-Ezzahra Hazmiri, Omar Oulghoul, Hanane Rais

**Affiliations:** 1 Department of Pathology and Laboratory Medicine, Mohammed VI University Hospital, Marrakech, MAR; 2 Department of Pathology, Mohammed VI University Hospital, Marrakech, MAR; 3 Department of Pathology, Faculty of Medicine and Pharmacy of Marrakech, Marrakech, MAR; 4 Department of Otorhinolaryngology, Mohammed VI University Hospital, Marrakech, MAR

**Keywords:** biphasic synovial sarcoma, fluorescence in situ hybridization (fish), laterocervical tumefaction, parotid region mass, pediatric, synovial sarcoma, translocation

## Abstract

Synovial sarcoma (SS) is a malignant mesenchymal proliferation most commonly found near the large joints and bursae of the lower extremities. It is rarely found in the head and neck region. The objective of our study is to report a female infant with SS of the right parotid gland. She did not present any particular pathological history and presented with a right laterocervical tumefaction rapidly increasing in size. Clinical examination showed a non-pulsatile, non-tender, and firm mass in the right parotid region measuring 7 cm x 5 cm with no local signs of aggression. Facial CT and MRI scans showed a lesion centred on the right parotid region, which was locally aggressive and displaced adjacent structures without invading them. Microscopic examination of the surgical biopsy revealed a spindle cell tumor proliferation, heterogeneous in cell density, not very atypical, with the presence of a few tubes and clusters of epithelial cells that resembled the ducts and acini of the normal parotid gland. After an immunohistochemical study, the diagnosis made was that of a SS with a double fusocellular and epithelial component. Molecular confirmation was obtained by searching for the SS18 gene translocation characteristic of synovial sarcoma using the Fluorescence in Situ Hybridization (FISH) technique, the result of which was positive.

SS is an aggressive type of sarcoma and is considered the leading cause of non-rhabdomyosarcoma soft tissue sarcomas in children and adolescents. Diagnosis is obtained by histological and immunohistochemical analysis of the tumor, complemented by a search for the specific t(X;18) translocation, found in over 90% of cases. The treatment of SS differs according to age. The use of perioperative chemotherapy followed by surgery and then radiotherapy is more common in pediatrics and may improve the prognosis and reduce the risk of recurrence and metastasis.

## Introduction

Synovial sarcoma (SS) is a malignant mesenchymal proliferation most commonly found near the large joints and bursae of the lower extremities. It is rarely found in the head and neck region [1] and accounts for 8-10% of soft tissue sarcomas in children and adolescents. It is the second most common type of malignant mesenchymal tumor after rhabdomyosarcoma [2]. Diagnosis is obtained by histological and immunohistochemical analysis of the tumor, supplemented by the search for the specific t (X; 18) translocation, found in more than 90% of cases [3]. Treatment modalities differ according to age, with the use of perioperative chemotherapy being much more frequent in pediatrics, with a therapeutic strategy close to the recommendations for the treatment of rhabdomyosarcomas [4]. The main prognostic factors reported in pediatric series are tumor size, primary site, and quality of surgical resection [5,6]. The aim of this paper is to report the case of a female infant with SS of the right parotid gland.

## Case presentation

**Figure 1:**
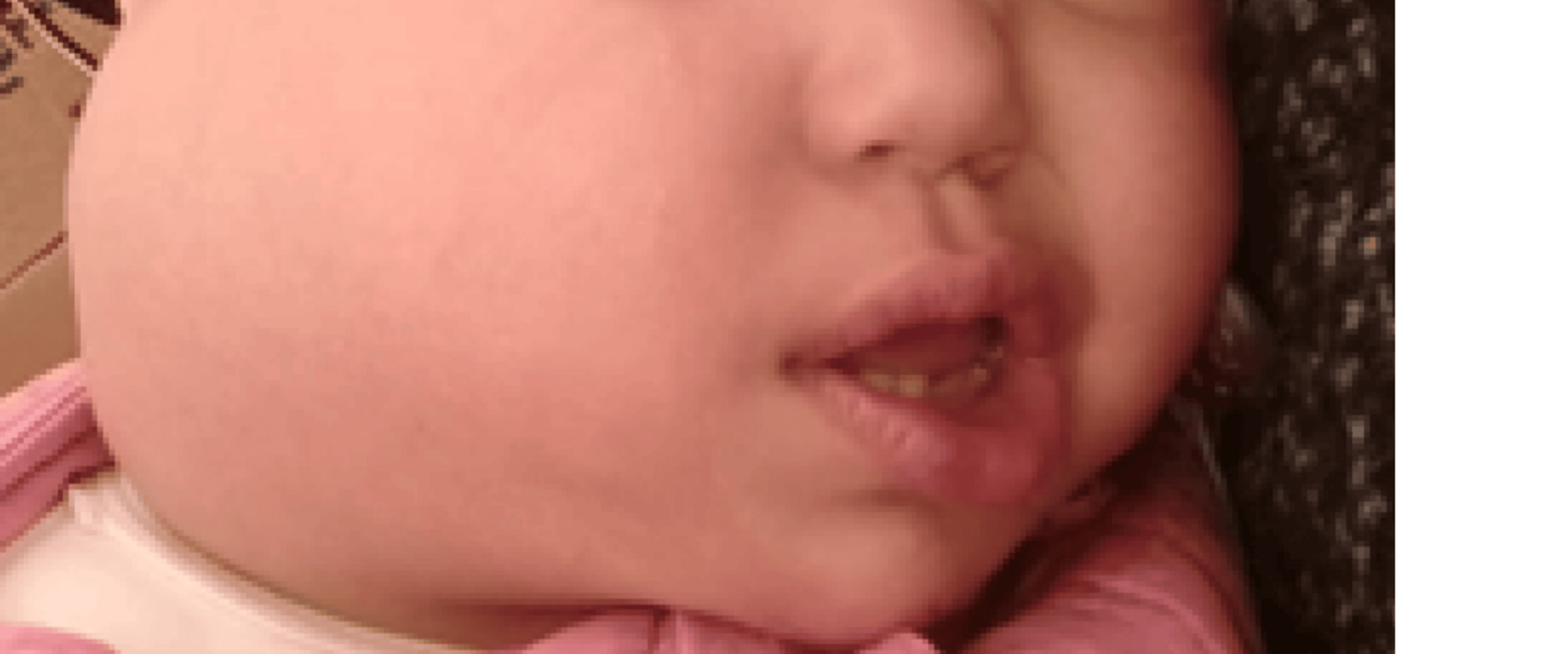
Right cervical mass Non-pulsatile, non-tender and firm mass in the right parotid region, elevating the ear lobule and measuring 7 cm x 5 cm, with no signs of local aggression

We report the case of a 10-month-old female infant with no specific pathological history. She was admitted to the Otorhinolaryngology (ORL) department for a rapidly increasing right latero-cervical tumefaction (evolving over four months prior to admission) without deterioration of the general condition. Clinical examination revealed a non-pulsatile, non-tender and firm mass in the right parotid region, elevating the ear lobule and measuring 7 cm x 5 cm (Figure 1). 

**Figure 2:**
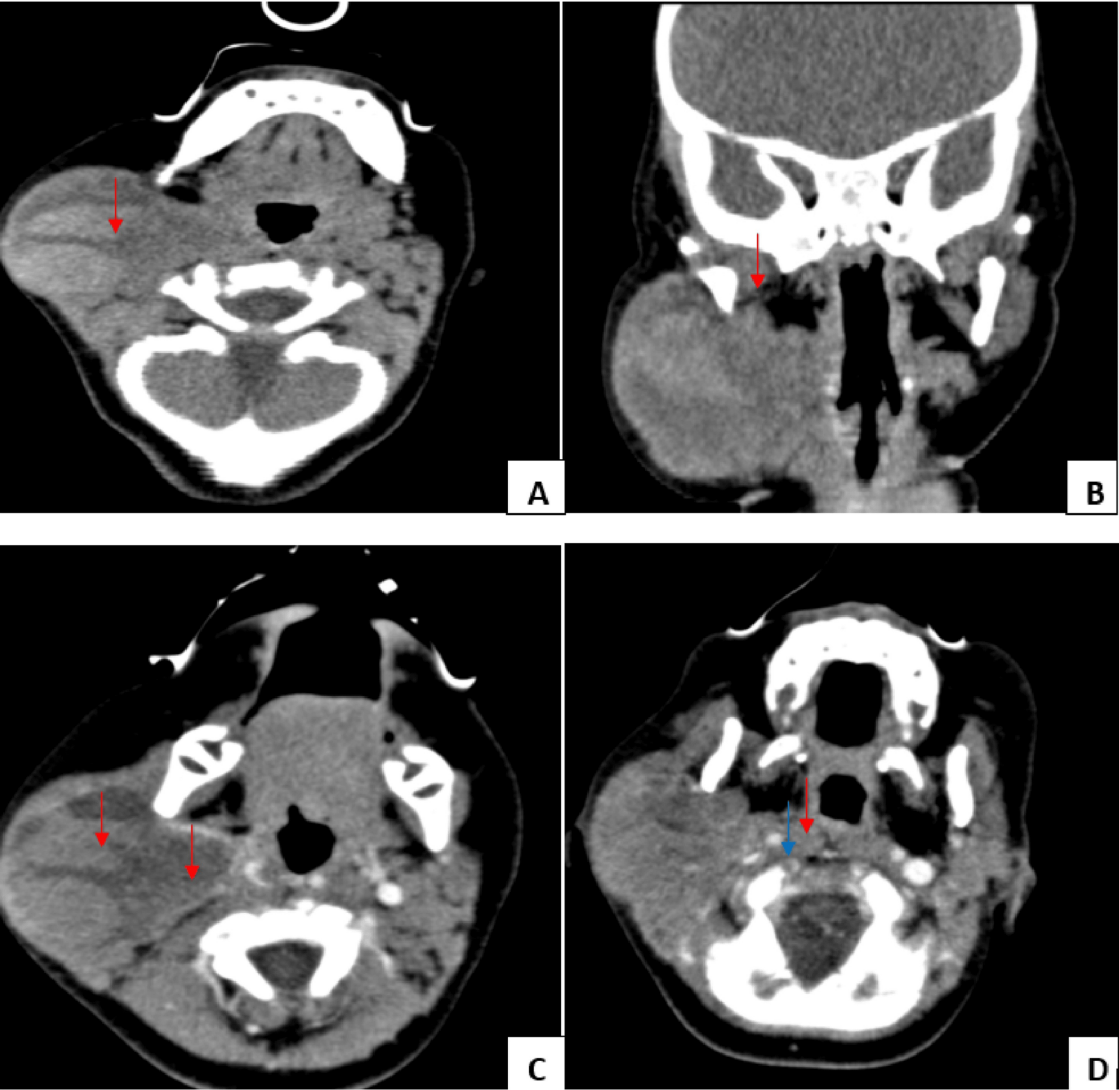
Facial CT scan A: Lesional process centered on the right parotid region (red arrow), well limited, of heterogenous density, with spontaneously hyperdense areas (hemorrhagic) delimiting foci of necrosis B: The process comes into contact with the temporomandibular joint (red arrow). C: After injection of the contrast agent (CA), this tumoral process discreetly enhances, delimiting foci of necrosis (red arrow). D: The lesional process encompasses the internal carotid artery, which remains of normal caliber and permeable (red arrow), and pushes back the internal jugular vein, which also remains permeable (blue arrow).

This mass showed no signs of local aggression, notably, no facial paralysis or trismus, no cervical adenopathy, and no obstruction or bleeding through the Stenon duct or obvious skin infiltration. Facial CT scan found a lesional process centered on the right parotid region, well-limited, and of heterogeneous density. It was the site of spontaneously hyperdense (hemorrhagic) areas delimiting the foci of necrosis and discreetly enhanced after injection of contrast agent. It encompassed the internal carotid artery, which remained of normal caliber and permeable, and pushed back the internal jugular vein, which also remained permeable. Above, this process came into contact with the temporomandibular joint (TMJ) and discreetly displaced the anterior wall of the external auditory canal (EAC) without lysis of bone or cartilage. Internally, it was locally infiltrative with partial filling of the prestylien, retrostylien, and right parapharyngeal space, along with discrete compression of the anterior wall of the EAC. Further down, it filled the prevertebral space opposite the C1, and posteriorly infiltrated the sternocleidomastoid muscle in places (Figure 2).

**Figure 3:**
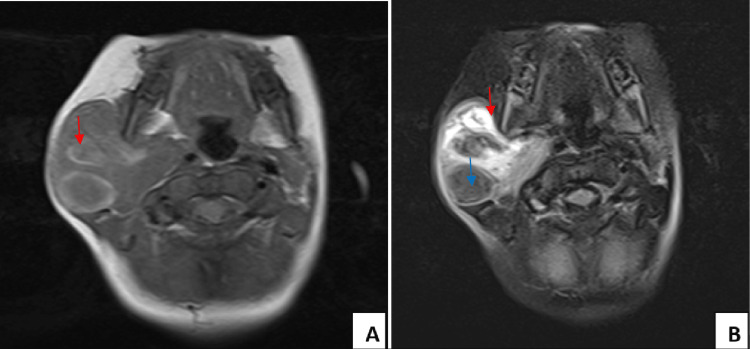
Facial MRI A: T1 sequence: Right latero-cervical lesional process centered on the right parotid region, fairly well limited, with a heterogenous hyposignal intensity on T1 weighted image. B: T1 sequence after injection of gadolinium: intensely and heterogeneously enhanced after injection of godalinium, delimiting large areas of necrosis (hyposignal T1; blue arrow) and stigmata of hemorrhage (hypersignal T1; red arrow).

MRI of the face showed a right laterocervical lesion centered on the right parotid region. It was fairly well limited, with a heterogeneous hyposignal intensity on T1-weighted, intensely and heterogeneously enhanced after injection of godalinium, delimiting large areas of necrosis (T1-hyposignal) and stigmata of hemorrhage (T1-hypersignal) (Figure 3).

A surgical biopsy was performed and sent to the pathological anatomy and cytology department of the Mohammed VI University Hospital in Marrakech. Three biopsy fragments were obtained, measuring 1.2 cm, 0.7 cm and 0.5 cm, respectively. They were included in their entirety. Six blank slides were requested for possible immunohistochemical study.

**Figure 4:**
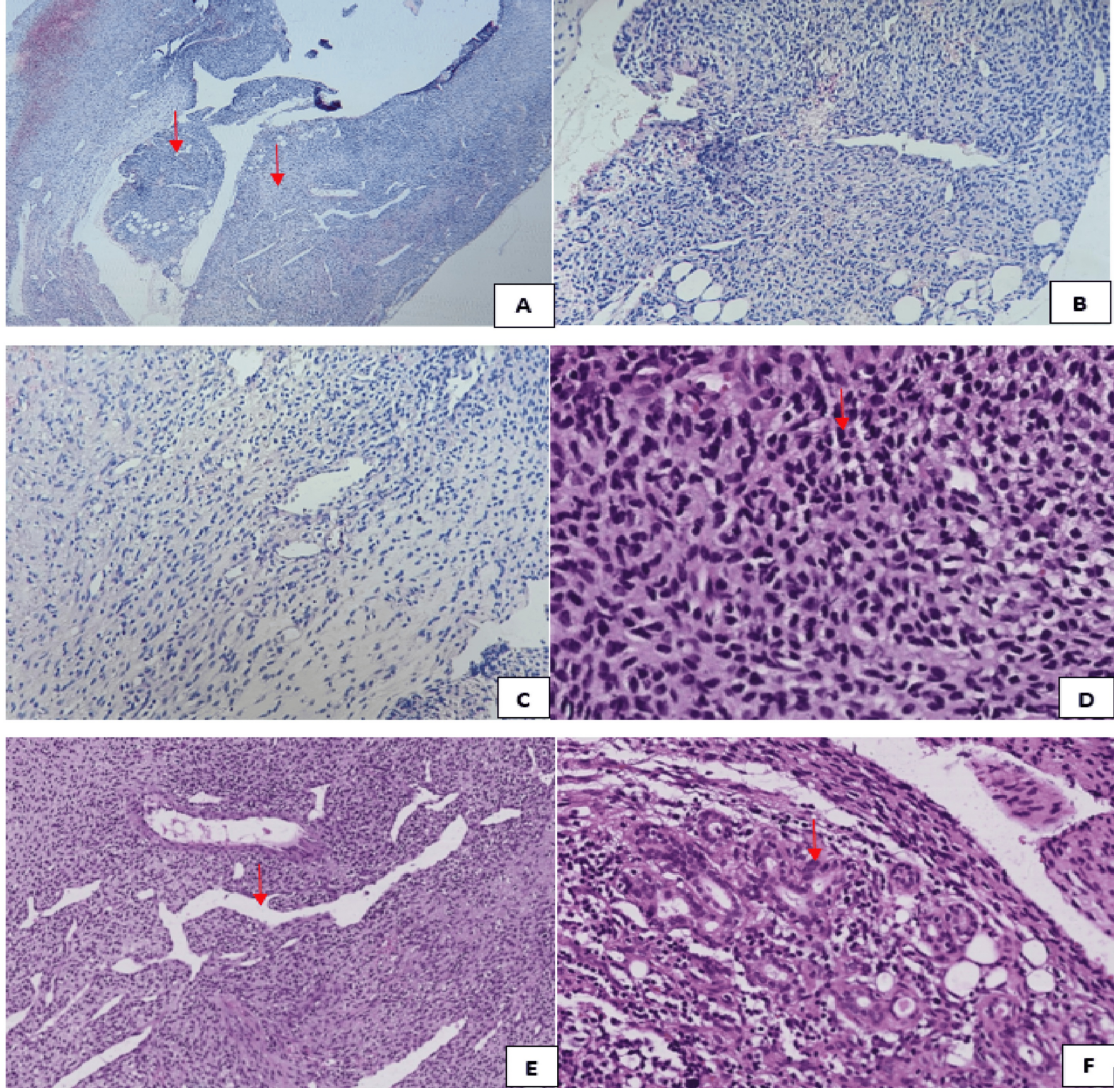
Microscopic examination A: Spindle cell proliferation of heterogenous cell density (Hematoxylin and Eosin (HE) staining, magnification x 4). B: Foci of high cell density (HE staining, magnification x 20). C: Foci of decreased cell density (HE staining, magnification x 20). D: The tumor cells are monomorphic, often short and overlapped in places (red arrow), with oval, hyperchromatic nuclei and some abnormal mitoses. The cytoplasm is sparse and eosinophilic (HE staining, magnification x 40). E: The stroma is fibro-collagenous, with a hemangiopericytic vascularization (HE staining, magnification x 20). F: A few tubes of medium sized epithelial cells with round, hyperchromatic nuclei. Their cytoplasm is of medium abundance and eosinophilic, clarified in places (HE staining, magnification x 40).

On microscopic examination, it showed a fibro-adipose tissue dissociated by a spindle cell tumor proliferation, of heterogeneous cell density, mostly high. It was arranged in diffuse layers. The tumor cells were monomorphic, often short and overlapping in places. They had oval, hyperchromatic nuclei with abnormal mitoses estimated at two mitoses/10 fields at high magnification. The cytoplasm was sparse and eosinophilic. Alongside this spindle cell proliferation, the histological examination showed the presence of a few tubes and clusters of epithelial cells which resembled the excretory ducts and acini of the normal parotid gland. The latter were of medium size, with round, hyperchromatic nuclei. Their cytoplasm was moderately abundantly eosinophilic and clarified in places. The stroma was fibro-collagenous in the hypo-cellular areas and had a rich hemangio-pericyte vascularization (Figure 4).

The main diagnoses to consider when it comes to pediatric spindle cell proliferation are: (1) rhabdomyosarcoma, being the most frequent malignant pediatric mesenchymatous tumor; (2) SS, which is the first non-rhabdomyosarcoma pediatric sarcoma; (3) infantile fibrosarcoma (IFS), which is a spindle cell proliferation of high cell density that affects children before the age of two years; and (4) malignant peripheral nerve sheath tumor that remains rare in children.

**Figure 5:**
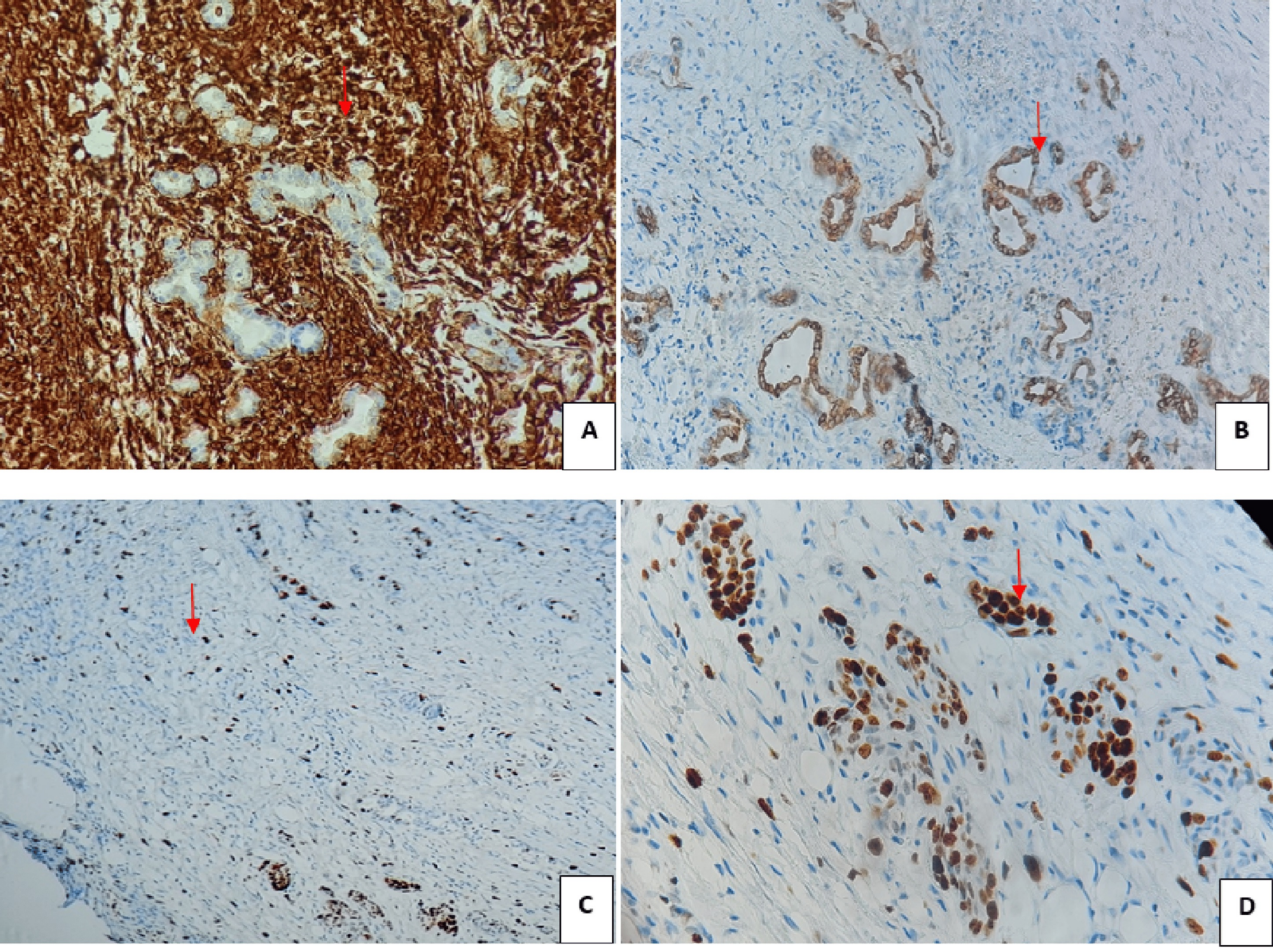
Immunohistochemistry results A: Intense and diffuse cytoplasmic expression of anti-vimentin antibody (clone: V9) in spindle cell (red arrow). B: Moderate and diffuse membrane expression of epithelial cells (clusters and tubes) of the anti-epithelial membrane antigen (EMA) antibody (Clone E29, Dako) (red arrow). C: Intense nuclear expression of 35% of fusocellular tumor cells of the Ki-67 antibody (Clone MIB1) (red arrow). D: Moderate to intense nuclear expression of 35% of epithelial cells of the Ki-67 antibody (Clone MIB1) (red arrow).

An immunohistochemical study was performed. The latter revealed an absence of expression of tumor cells of the anti-desmin, anti-myogenin and anti-PS100 antibodies. A diffuse and intense expression of tumor cells of the anti-vimentin antibody was revealed. The anti-epithelial membrane antigen (EMA) antibody was negative in spindle cell proliferation but moderately and diffusely expressed in the clusters and tubes described above. The Ki-67 proliferation index was 35% in both the spindle cell and epithelial structures, suggesting that this was a SS with both spindle cell and epithelial components (Figure 5).

**Figure 6:**
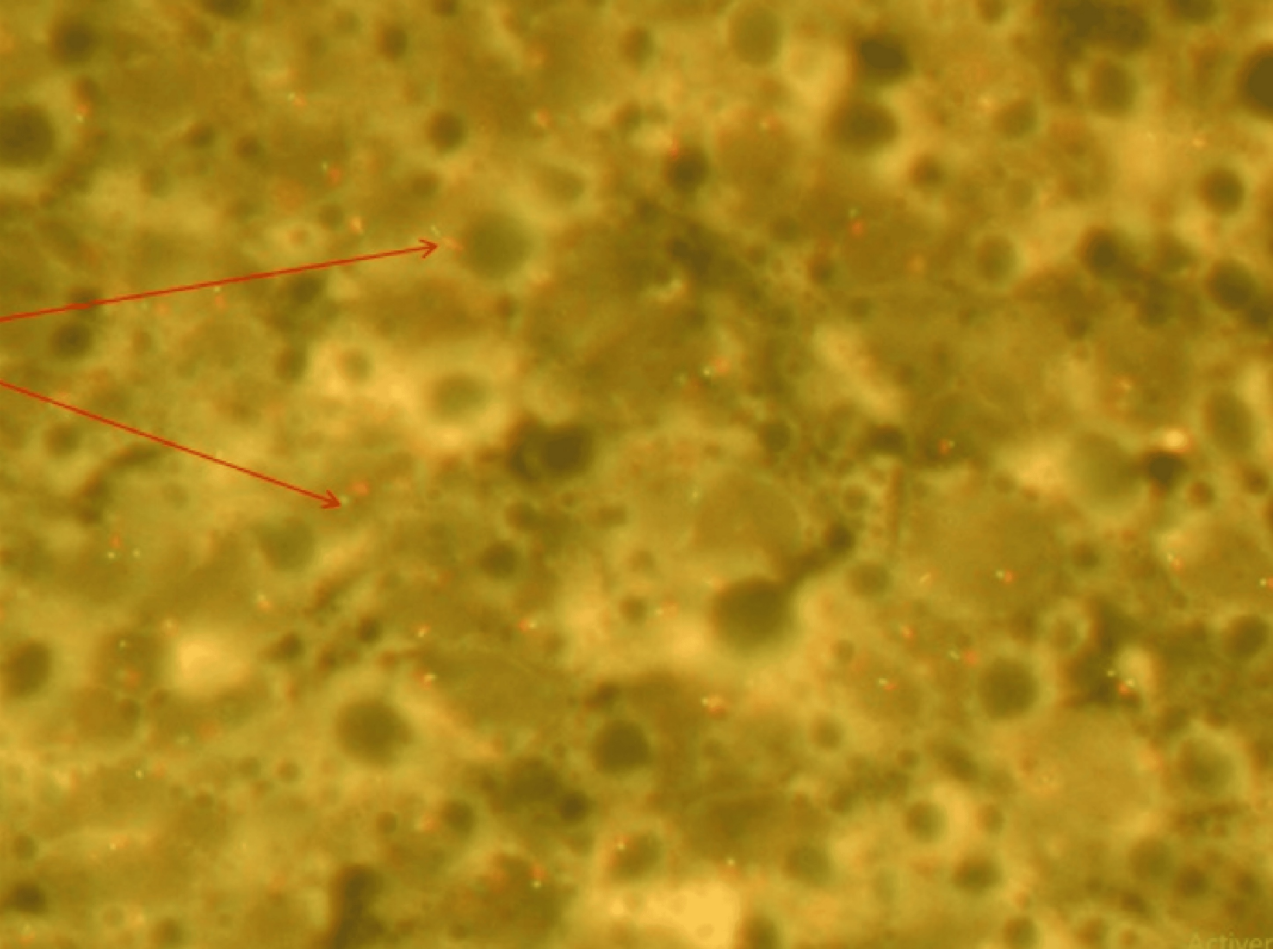
Fluorescence in situ hybridization (FISH) Demonstration of the chromosomal translocation t (X; 18) (p11; q11) by FISH: 30% of the tumor cells show a translocation of the SS18 gene (red arrow).

For diagnostic confirmation, molecular biology testing for the SS18 gene translocation, characteristic of SS, was performed by Fluorescence in Situ Hybridization (FISH) using the SPEC SS18 Dual Color Break Apart Probe (Zytovision GmbH, Germany). The result was positive, with 30% of the tumor cells showing the SS18 gene translocation (Figure 6).

The diagnosis was therefore that of a biphasic SS of the parotid gland. Following a multidisciplinary discussion, the therapeutic strategy consisted of neoadjuvant chemotherapy (six cycles) based on ifosfamide and doxorubicin, followed by surgical resection of the tumor. Radiotherapy was excluded due to the major risk of irreversible sequelae in infants, particularly craniofacial growth retardation and potential cerebral damage.

Surgical excision was subtotal because of the persistence of a residual tumor in the retrostyloid space, adherent to the internal carotid artery. A follow-up cervical MRI was performed nearly two months after surgery and revealed a persistent, relatively well-defined area of residual infiltration in the right laterocervical region, centered on the right parotid space, measuring 26 × 45.5 × 58 mm, with associated infiltration of the adjacent soft tissues.

In this context, adjuvant chemotherapy (four additional cycles) based on ifosfamide and doxorubicin was indicated, along with regular follow-up.

## Discussion

SS is defined by the World Health Organization (WHO) as a monomorphic spindle cell sarcoma with variable epithelial differentiation and characterized by a specific SS18-SSX 1/2/4 fusion gene [7]. It can occur at any age but is most commonly observed in young adults (15-40 years). It accounts for 17.6% of cases in children and young adults (0-19 years), with a mean age of onset of 13 years [1-3]. SS represents 10-15% of soft tissue sarcomas in children and adolescents and is considered the leading cause of non-rhabdomyosarcoma soft tissue sarcomas [6]. It affects male patients 3.7 times more frequently than female patients [8].

The anatomical distribution of SS varies with age. In adults, it primarily arises in the deep soft tissues of the extremities, particularly the lower limbs, and near large joints (70%), followed by the trunk (15%) and the head and neck region (7%). Primary involvement of the parotid gland is extremely rare (0.5%). In children, the limbs are also the most common site (65%). Atypical locations, including the head and neck (20%), the trunk (15%), and the parotid gland (3%), are slightly more frequent in pediatric patients than in adults [7].

Clinically, SS typically presents as a well-defined, variably fixed soft tissue mass, often small, particularly in the head, neck, or hands. It is generally painless, and its growth is usually slow, progressing over several months. Cervical involvement may be associated with facial paralysis or cervical lymphadenopathy [9]. This deceptively benign presentation often contributes to a significant diagnostic delay, with one study reporting a mean delay of 98 weeks [8-9].

The initial workup includes Doppler ultrasound of the tumor mass to assess its contours and internal characteristics. The lesion appears solid and homogeneous when small, but becomes heterogeneous with semi-liquid and necrotic components as it increases in size. Vascularization is typically present and detectable on ultrasound, though its degree varies [6]. However, the precise relationship of the tumor to adjacent bone and neurovascular structures is best evaluated with magnetic resonance imaging (MRI). MRI is essential for the assessment of any soft tissue mass prior to surgery, but it does not allow for a definitive diagnosis of SS because it lacks a pathognomonic appearance. It can nonetheless help rule out certain differential diagnoses that may be suggested by ultrasound. The MRI features may be suggestive of SS when the lesion displaces surrounding structures rather than invading them, as was observed in our case. Most of these tumors appear heterogeneous, particularly when larger than 5 cm, with extensive hemorrhagic and necrotic areas often associated with a cystic component [10]. Computed tomography (CT) remains useful for detecting intratumoral calcifications and evaluating bone erosion [11].

Ideally, the diagnosis of SS is established through histological analysis following a tumor biopsy. Although biopsy can be performed surgically, image-guided percutaneous needle biopsy is increasingly preferred [9]. On macroscopic examination of the surgical specimen, SS typically appears as a well-defined but non-encapsulated tumor, measuring between 3 and 10 cm. It is usually round and may be multinodular, with a grayish-white or yellowish-pink cut surface. Its consistency ranges from soft to firm depending on cellularity and collagenization. Cystic, hemorrhagic, and necrotic changes may also be present [7]. Calcifications are observed in approximately one-third of cases [9].

According to the WHO international classification, two classic histological subtypes of SS are described: the monophasic subtype-the most common, particularly in adults-composed exclusively of spindle cells; and the biphasic subtype, which contains both spindle cells and differentiated glandular epithelial cells in varying proportions [7-9].

The experience of the International Society of Paediatric Oncology (ISOP), involving 49 pediatric cases of localized SS diagnosed before the age of 18 between 1995 and 2003, showed that 55% of cases were monophasic and 45% biphasic [12]. The prognostic significance of these subtypes has not been established in either pediatric or adult populations [13].

Morphologically, SS typically consists of multiple nodules separated by fibrous septa, showing alternating areas of densely cellular, basophilic regions and less cellular zones. In some cases, the architecture is fasciculated and hypercellular, with a reduced stromal component. Tumor cells are spindle-shaped, monomorphic, and relatively short, with an oval, hyperchromatic nucleus, uniformly dispersed chromatin, and inconspicuous nucleoli, along with occasional mitotic figures. The cytoplasm is scant, eosinophilic, and poorly demarcated, resulting in a high nuclear-to-cytoplasmic ratio and frequent cell overlap [7-14], features that should initially raise suspicion for SS. Collagen deposition, cystic changes, or calcifications may also be present [15].

The diagnosis of biphasic SS requires the identification of an epithelial component arranged in clusters, cords, or glandular structures in addition to the spindle cell component previously described. The extent of the glandular component is highly variable. The epithelial cells are cuboidal or columnar, with round or ovoid nuclei, and their cytoplasm is generally eosinophilic and more abundant than that of the spindle cell component [7]. In rare cases, the glandular component may predominate and mimic adenocarcinoma; however, at least a small proportion of spindle cells is always present [14].

More rarely, these tumors contain a poorly differentiated component composed of small round, spindle, or epithelioid cells showing marked nuclear atypia and high mitotic activity [7]. These poorly differentiated forms of SS are most often diagnosed in adults - particularly older adults - and appear to be absent in children [5]. Only one study of SS diagnosed in adulthood has demonstrated a significant survival difference based on histological subtype, with a poorer prognosis for tumors containing a poorly differentiated component [13].

Immunohistochemical analysis of SS shows variable expression of epithelial markers such as EMA and cytokeratins (the latter often more focal in the monophasic subtype). Only the spindle cell component expresses mesenchymal markers such as vimentin. Focal S100 protein expression is detectable in approximately 40% of SS cases, while CD99 is expressed in about 50%, sometimes with membranous staining similar to that observed in Ewing sarcoma. Nuclear staining for the transcriptional corepressor TLE1 is seen in roughly 80% of cases, although this marker can also be expressed in tumors with overlapping histological features, such as malignant peripheral nerve sheath tumors (MPNSTs) or solitary fibrous tumors [8-11].

Characteristically, in more than 90% of cases, SS harbors the specific translocation t(X;18)(p11.2;q11.2), resulting in the fusion of the SYT gene on chromosome 18 with the SSX1 gene (in two-thirds of cases), the SSX2 gene (in fewer than one-third of cases), or, much more rarely, the SSX4 gene, all located on the X chromosome [14].

The monophasic form of SS can be challenging to diagnose because it may morphologically resemble IFS, spindle cell rhabdomyosarcoma (RMS), MPNST, or Ewing sarcoma/primitive neuroectodermal tumor (PNET) [15-18].

IFS occurs primarily in infants and young children. Histologically, it is characterized by bundles of ovoid and spindle-shaped cells, often arranged in a herringbone pattern. At the molecular level, it is defined by the t(12;15)(p13;q25) translocation resulting in the ETV6-NTRK3 fusion, which is absent in SS. Moreover, IFS generally does not express epithelial markers (cytokeratins/EMA), which may be focally expressed in SS [15].

Morphologically, RMS may also mimic monophasic spindle cell SS. However, immunohistochemical positivity for myogenin and MyoD1 strongly supports the diagnosis of RMS and effectively rules out SS [16].

MPNSTs can be morphologically confused with SS because of their spindle cell architecture. They are often clinically associated with neurofibromatosis type 1. Immunohistochemically, expression of S100 (variable) and SOX10 may suggest a neural origin, whereas epithelial markers are rarely detected in MPNSTs. In contrast, nuclear expression of TLE1 and the detection of the SS18-SSX fusion are highly suggestive of SS [17].

Ewing sarcoma is characterized by a small, round, non-spindle cell morphology and strong membranous expression of CD99, which may occasionally overlap with the immunohistochemical profile of SS. However, the absence of epithelial differentiation and the presence of the t(11;22)(q24;q12) translocation involving the EWSR1 gene support a diagnosis of Ewing sarcoma rather than SS [18].

Thus, although SS may present suggestive morphological and immunohistochemical features, the combined use of an extended immunohistochemical panel (including EMA, cytokeratins, myogenin, MyoD1, S100, SOX10, and TLE1) together with molecular confirmation of the SS18-SSX fusion remains essential to exclude the main differential diagnoses [15-18].

At the molecular level, the fusion transcript characteristic of SS is consistently present in both the primary tumor and its metastases. Its detection by in situ hybridization or reverse transcription polymerase chain reaction (RT-PCR) serves as a diagnostic marker that should be routinely used in clinical practice to confirm the histological diagnosis [19,20].

A strong association between the type of fusion transcript and the histological subtype has been reported. Most SYT-SSX2 tumors exhibit a monophasic phenotype, whereas nearly all tumors with biphasic histology harbor an SYT-SSX1 transcript [19,20]. However, more recent studies have not demonstrated a correlation between the histological subtype of SS and patient prognosis [12-14].

The precise prognostic value of the fusion transcript type has rarely been prospectively evaluated in pediatric and adolescent SS. Only one study by Ferrari et al. [13], which analyzed outcomes in 138 pediatric SS patients in Europe between 2005 and 2012, found that the fusion transcript type did not influence prognosis. In contrast, several retrospective studies in adults suggest that the presence of the SYT-SSX1 transcript may be associated with shorter metastasis-free survival [15], or that patients with SYT-SSX2-positive tumors may experience significantly better overall survival [3].

Compared to rhabdomyosarcomas, other soft tissue sarcomas in children and adolescents, including SS, are classically considered less chemosensitive and associated with a less favorable prognosis, with five-year survival rates ranging from 60% to 84% [12-17].

Several prognostic factors have been identified, although prospective studies are needed to clarify their true impact. In children, younger age is associated with a better prognosis [19,20]. Tumor size (≥ 5 cm in children and adolescents) is the main independent prognostic factor, as it correlates with an increased risk of metastatic progression. Tumor location and depth of infiltration are also associated with overall survival [13]; tumors located in the limbs have a more favorable prognosis than those in axial sites. The extent of disease at diagnosis is another key prognostic factor, with metastatic cases showing an estimated five-year survival rate of only 13%. The quality of surgery-specifically, the completeness of excision-is linked to the risk of local (but not metastatic) recurrence [20].

Histological prognostic factors include the presence of a poorly differentiated component (anaplasia and/or tumor necrosis), mitotic activity ≥ 10/HPF or a high Ki-67 proliferation index, a high tumor grade according to the French National Federation Against Cancer (Fédération Nationale des Centres de Lutte Contre le Cancer or FNCLCC) system [9], and potentially the presence of the SYT-SSX1 transcript, which may be associated with shorter metastasis-free survival [19].

Treatment strategies for SS vary with age, with optimal local control being essential, particularly in adults, where management relies primarily on surgery and radiotherapy. Perioperative chemotherapy is more frequently used in pediatric patients, following an approach similar to that employed for rhabdomyosarcoma [9,13,20].

The local recurrence rate of SS is strongly correlated with the adequacy of local disease control. Studies by Sultan et al. have shown that, in addition to tumor size and positive margins, the absence of radiotherapy is a significant risk factor for local recurrence. When administered preoperatively, radiotherapy may facilitate resectability and reduce the risk of metastasis [20]. The rapid recurrence observed in our case can be attributed to incomplete surgical resection and the absence of perioperative radiotherapy, which might have improved the patient’s prognosis.

## Conclusions

SS is an aggressive type of sarcoma and is considered the leading cause of non-rhabdomyosarcoma soft tissue sarcomas in children and adolescents. SS of the cervical location should be considered when there is a progressively evolving tumefaction of the parotid region seen on MRI, that displaces adjacent structures without invading them. The immunohistochemical profile of this type of sarcoma is suggestive, but molecular confirmation is still required by searching for the t (X; 18) (p11.2; q11.2) using the FISH technique.

Therapeutic management of SS varies according to age. In our case, the therapeutic decision consisted of neoadjuvant chemotherapy followed by surgical resection, which was incomplete due to adhesion to the internal carotid artery, and subsequently adjuvant chemotherapy without radiotherapy. This treatment approach may improve prognosis while reducing the risk of metastatic dissemination of the tumor. Key genetic alterations involved in the genesis of SS tumors could be the target of new therapies.
